# Complete Chloroplast Genome Sequence of the Western Poison Oak, Toxicodendron diversilobum (Anacardiaceae), from California

**DOI:** 10.1128/mra.01279-22

**Published:** 2023-02-16

**Authors:** Laura I. Huitron Vazquez, Perla E. Aviles, Samantha A. Bailon, Abner G. Cabanillas, Andrea Fernandez, Juan I. Galarza, Brianna Guerrero, Araceli B. Hernandez, Daniel Hernandez, Khegan Jarrett, Tong Li, Francisco J. Maravillo, Magdalena Moreno, Azalea Perez, Nathan A. Rosales, Hunter F. Ruegg, Joel Valdez, Kyla Mae Bravo, Vidal L. Chávez, Daisy I. Diaz, Daniela Enriquez, Edgar L. Martinez, Jesus Mendoza Padilla, Jose Meza, Scott V. Nelson, Crystal Quintero-Ahumada, Adriene Mariah Ramirez, Jeffery R. Hughey

**Affiliations:** a Division of Mathematics, Science, and Engineering, Hartnell College, Salinas, California, USA; University of Strathclyde

## Abstract

Here, we present the complete chloroplast genome sequence of Toxicodendron diversilobum, western poison oak, from Pacific Grove, California. The genome is 159,543 bp in length, contains 133 genes, and has a high level of gene synteny to other species of *Toxicodendron*.

## ANNOUNCEMENT

Toxicodendron diversilobum (Torr. & A. Gray) Greene, western poison oak, was originally described from material collected by the botanist and explorer David Douglas from Fort Vancouver, Washington, USA (1–3). The species is naturally distributed from British Columbia, Canada, to Baja California, Mexico, where it grows as a vine or shrub inhabiting canyons, slopes, chaparral, and oak woodland communities (4, 5). All tissues of T. diversilobum contain the toxin urushiol, which causes severe dermatitis in about 80% of humans (6, 7). Five complete *Toxicodendron* chloroplast genomes have been sequenced (8–12); however, the western poison oak has not been analyzed. In this study, we characterized the complete chloroplast genome of T. diversilobum to contribute to its bioinformatics.

The specimen analyzed here was collected from Pacific Grove, California (36°37′10.9″N, 121°55′41.6″W), and deposited at Hartnell College under voucher number HCC 271. Fresh leaf tissue was macerated with a mortar and pestle, and DNA was extracted using the DNeasy blood and tissue kit (Qiagen) following the manufacturer’s protocol with two modifications; the binding step was reduced to 4,000 × *g* for 3 min, and the DNA was eluted in 40 μL Tris-acetate-EDTA (TAE) buffer after 7 min of incubation (13). The 150-bp paired-end library was constructed with the NEBNext Ultra II DNA library preparation kit (New England BioLabs) and sequenced by Novogene on an Illumina NovaSeq 6000 system. The analysis generated 13,641,742 raw reads. The reads were filtered using the default BBDuk settings in Geneious Prime v2019.1.3 (Biomatters Limited). The chloroplast genome was assembled *de novo* using all filtered reads with the default settings in Geneious Prime and RepeatFinder v1.0.1 (14). This process yielded 80,852 contigs, with an *N*_50_ value of 409 and a GC content of 37.2%. A single draft chloroplast genome contig with overlapping ends and 1,291× coverage was identified using the Map to Reference function in Geneious Prime and the reference Toxicodendron vernicifluum cultivar Dahongpao (GenBank accession number NC_046700) (8). The final chloroplast genome was circularized by removing the overlapping ends and manually adjusting the start position to conform to the reference sequence. The annotation was completed using the default settings in GeSeq v2.03 (15), followed by manual adjustment of the start and stop positions according to the NCBI open reading frame (ORF) finder and Sequin v15.5 (16).

**FIG 1:**
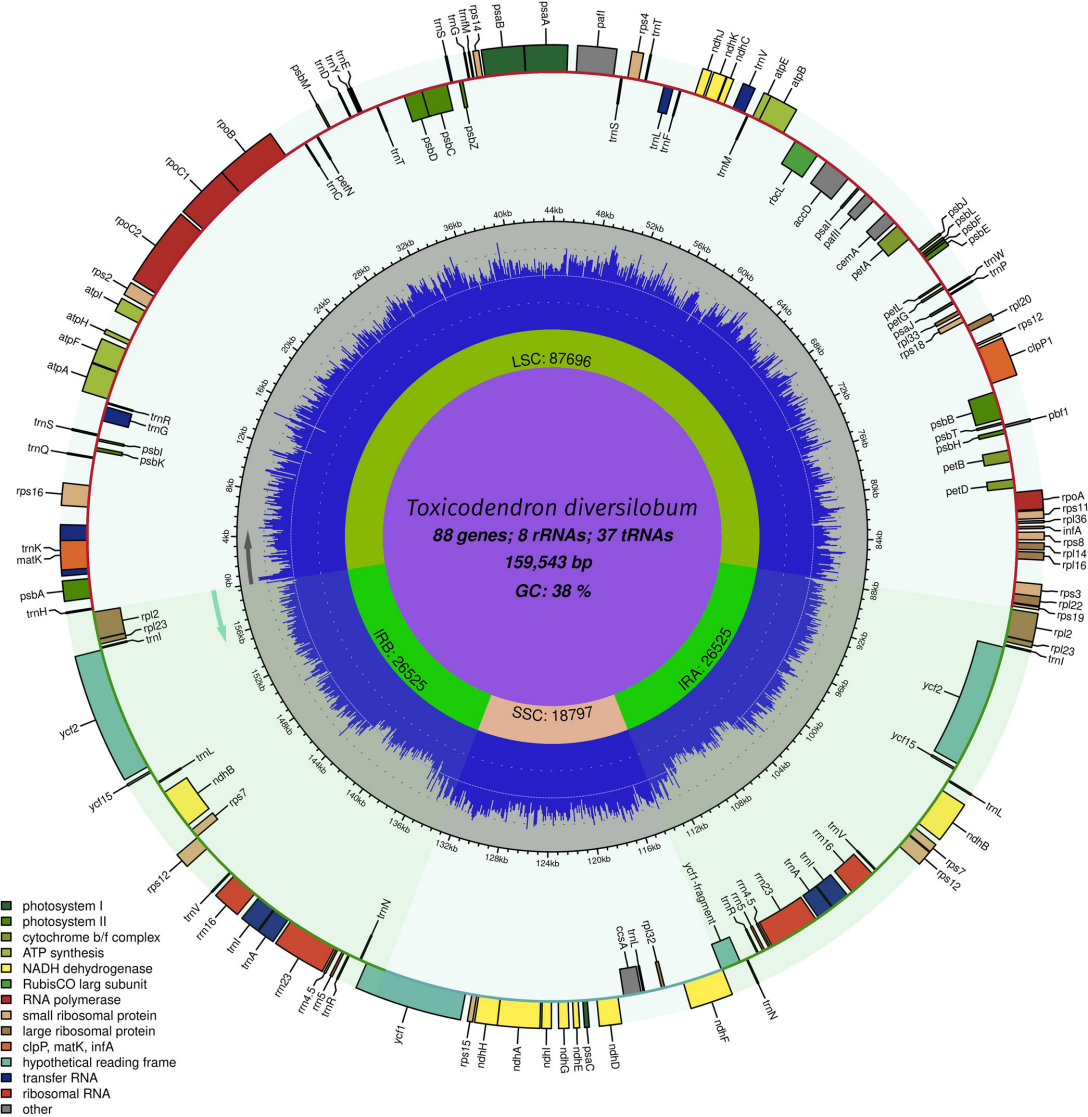
Complete chloroplast genome of Toxicodendron diversilobum. The genome was annotated using GeSeq (15), the NCBI ORF finder, and Sequin v15.5 (16) and mapped with Chloroplot v0.2.4 (18). The innermost ring identifies the LSC, SSC, and two IRs. The next ring displays the GC content and direction of transcription, as indicated by the two arrows. The final ring shows the genes. Genes transcribed clockwise are on the inside, while those transcribed counterclockwise are on the outside. The color coding corresponds to genes of different groups, as listed in the key at the bottom left.

The complete circular chloroplast genome of T. diversilobum is 159,543 bp in length and displays a characteristic flowering plant quadripartite structure (17), possessing a large single-copy region (LSC), a small single-copy region (SSC), and two inverted repeats (IRs), with lengths of 87,696 bp, 18,797 bp, and 26,525 bp, respectively (Fig. 1). The GC content is 38.0%. The genome contains 133 genes, including 88 protein-encoding genes, 37 tRNA genes, and 8 rRNA genes. Sixteen of the genes contain one intron (*atpF*, *ndhA*, *ndhB* × 2, *rpl2* × 2, *rpoC1*, *rps16*, *trnA* × 2, *trnG*, *trnI* × 2, *trnK*, *trnL*, and *trnV*), and four contain two introns (*clpP*, *pafI*, and *rps12* × 2). Gene content and organization are consistent with other species of *Toxicodendron* (8–12). The chloroplast genome of T. diversilobum has 99.64% nucleotide identity to T. vernicifluum (GenBank accession number NC_046700), 99.55% to Toxicodendron griffithii (GenBank accession number NC_053916), 99.49% to Toxicodendron sylvestre (GenBank accession number MT211615), and 99.48% to Toxicodendron succedaneum (GenBank accession number MT211614).

### Data availability.

The complete chloroplast genome sequence of T. diversilobum is available in GenBank under accession number OP585546. The associated BioProject, SRA, and BioSample accession numbers are PRJNA902018, SRS15770361, and SAMN31746121, respectively. The chloroplast genomes referenced in the text were T. vernicifluum cultivar Dahongpao (GenBank accession number NC_046700), T. griffithii (GenBank accession number NC_053916), T. sylvestre (GenBank accession number MT211615), and T. succedaneum (GenBank accession number MT211614).
